# Postcontrast Fluid-Attenuated Inversion Recovery (FLAIR) Sequence MR Imaging in Detecting Intracranial Pathology

**DOI:** 10.1155/2020/8853597

**Published:** 2020-10-14

**Authors:** Ajit Mahale, Shaloo Choudhary, Sonali Ullal, Merwyn Fernandes, Sonali Prabhu

**Affiliations:** Department of Radiodiagnosis, Kasturba Medical College Mangalore, MAHE Dakshina Kannada, Karnataka 575001, India

## Abstract

**Background:**

Imaging sequences for detection of meningeal and parenchymal lesions are critical in intracranial pathology. Our study analysed FLAIR MRI sequence for evaluating postcontrast enhancement.

**Objectives:**

FLAIR imaging sequences have been used in evaluation of enhancement in the brain. We conducted a study of FLAIR imaging sequences to better delineate postcontrast enhancement.

**Materials and Methods:**

In this prospective hospital-based observational study, postcontrast T1 MTC and delayed postcontrast T2 FLAIR and T1 FLAIR images of 66 patients with intracranial pathology were assessed by experienced radiologists from November 2017 to November 2019.

**Results:**

28 cases of meningeal enhancement were identified in delayed postcontrast T2 FLAIR images. Low-grade gliomas included in the study showed postcontrast enhancement on postcontrast T1 MTC images. Multiple sclerosis lesions were better seen on postcontrast T1 FLAIR. In extraaxial lesions of 11 cases of meningioma, brighter enhancement was seen on delayed postcontrast T2 FLAIR images.

**Conclusion:**

We found that delayed postcontrast T2 FLAIR was better in detection of meningeal enhancement in infectious meningitis and in meningitis carcinomatosis than T1 MTC images. In delayed postcontrast T2 FLAIR images, intra-axial parenchyma lesions appeared more conspicuous or similar to T1 MTC images. Delayed postcontrast T1 FLAIR images provided better anatomic delineation of intra-axial lesions.

## 1. Introduction

The role of imaging sequences for more accurate detection of meningeal and parenchymal lesions is critical in diagnosis [1, 2]. Some studies performed in the last decade show that postcontrast T2 FLAIR is a useful imaging sequence for evaluation enhancement [3]. There are not many studies done to determine the utility of postcontrast T1 FLAIR in intracranial pathologies. The greatest advantage of CE FLAIR seems to be for detecting subtle abnormalities such as leptomeningeal carcinomatosis, where there is no mass effect^.^[4, 5]. We conducted a study to validate utilization of FLAIR sequences in delineating postcontrast enhancement.

## 2. Methods and Materials

In this prospective hospital-based observational study, postcontrast T1 MTC and delayed postcontrast T2 FLAIR and T1 FLAIR images of 66 patients—32 patients with meningeal enhancement (28 cases of infectious etiology and 4 with tumoral meningeal lesions-metastasis), 23 parenchymal enhancing lesions (7 patients with infection (5 cases tuberculomas and 2 toxoplasmosis), 2 patients with demyelination (multiple sclerosis), 6 patients with primary tumors (4 low-grade gliomas and 2 high-grade gliomas), and 8 patients with metastatic lesions), and 11 patients with extraaxial lesions(meningiomas)—were assessed by experienced radiologists during a study period from November 2017 to November 2019.

The study was approved by the institutional ethics committee. Data were collected from all age groups of patients who were referred to the department of radio diagnosis for MRI brain study with contrast, to rule out meningeal or parenchymal lesions.

Data were collected from the patients referred to the department of radio diagnosis, for MR imaging of the brain, in the teaching hospitals attached to Kasturba Medical College, Mangalore. MR examinations were performed on 1.5 Tesla (Magnetom Siemens Avanto).

Gadolinium dimeglumine (0.5 mmol/ml) was administered.

Sequential order of postcontrast sequences was as follows: T1W postcontrast sequence was run prior to delayed postcontrast FLAIR (fluid attenuation inversion recovery) sequences.

### 2.1. Scanning Parameters

Imaging parameters for delayed (10 min) postcontrast T2 FLAIR imaging:
    TR:9000
    TE:92
    TI:2500
    Slice thickness: 5 mm
Imaging parameters for postcontrast T1W FLAIR:
    TR:2000
    TI:860
    TE:59
    Slice thickness: 5 mm
Imaging parameters for postcontrast T1W MTC images:
    TR:741
    TE:17
    Slice thickness: 5 mm

### 2.2. Inclusion Criteria

Patients referred for gadolinium-enhanced brain imaging.

### 2.3. Exclusion Criteria

1. Patients with intracranial aneurysm clips.
2. Patients with intraorbital metal fragments, any electrically, magnetically, and mechanically activated implants (cardiac pacemakers, biostimulators, neurostimulators, cochlear implants, and hearing aids).
3. Patients having renal failure or raised creatinine (1.6 mg/dl) levels.

## 3. Results

**Figure 1:**
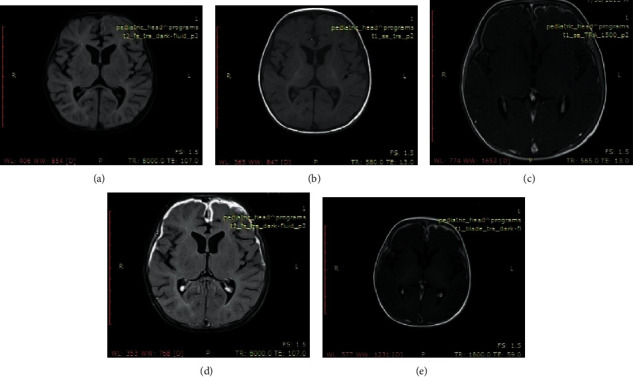
Case of meningitis: (a) unenhanced T2 FLAIR image; (b) unenhanced T1 image; (c) postcontrast T1 MTC image; (d) delayed postcontrast T2 FLAIR image; (e) delayed postcontrast T1 FLAIR image.

Out of the 28 cases with clinical diagnosis of leptomeningitis, in all of the 28 cases, meningeal enhancement was easily identified in delayed postcontrast T2 FLAIR images. In comparison, in postcontrast T1 MTC images, differentiation of meningeal enhancement from vascular enhancement was difficult and could have been missed easily. CSF studies were positive in all 28 cases. On the contrary, delayed postcontrast T1 FLAIR images demonstrated minimal meningeal enhancement in 26 cases out of the 28 cases of leptomeningitis, and the enhancement was inferior to T1 MTC images in all of these 28 cases (Figure 1).

Out of the 23 cases of intra-axial parenchymal lesions included in the study, in 2 cases, postcontrast T1 FLAIR was not done. In rest of 21 cases, delayed postcontrast T1 FLAIR was done, out of which 13 cases showed brighter and thicker enhancement of solid components and peripheral walls of the lesions. It provided better delineation of lesion. These 13 cases included 4 cases of solid cystic parenchymal mass lesions (2 primary and 2 metastatic), 2 cases of peripherally enhancing primary cystic tumors, 2 cases of cystic metastasis, 4 cases of infection (3 tuberculomas and 1 toxoplasmosis), and 1 case of demyelination (multiple sclerosis). In such cases, characteristics of the wall of peripherally enhancing tumors/infectious lesions were better defined by delayed postcontrast T1 FLAIR images as compared to postcontrast T1 MTC.

**Figure 2:**
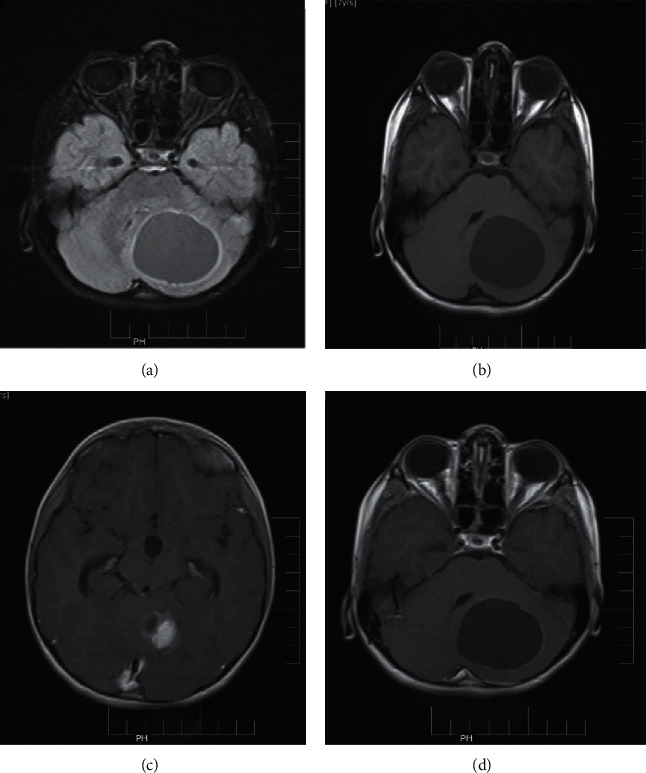
Pilocytic astrocytoma: (a) unenhanced FLAIR images; (b) unenhanced T1 images; (c, d) postcontrast T1 MTC.

In three out of four low-grade glioma included in the study, subtle postcontrast enhancement seen on postcontrast T1 MTC images could not be well appreciated on delayed postcontrast T2 FLAIR images due to the intrinsic FLAIR hyperintense nature of the lesions (Figure 2). Subtle enhancement noted in postcontrast T1 MTC images in all 4 low-grade gliomas was better delineated on delayed postcontrast T1 FLAIR images.

Postcontrast enhancement could not be well appreciated on delayed postcontrast T2 FLAIR images in two cases of multiple sclerosis as compared to postcontrast T1 MTC images. In patients with multiple lesions delayed T1 postcontrast FLAIR images picked up almost the same number of lesions as compared to postcontrast T1 MTC images.

**Figure 3:**
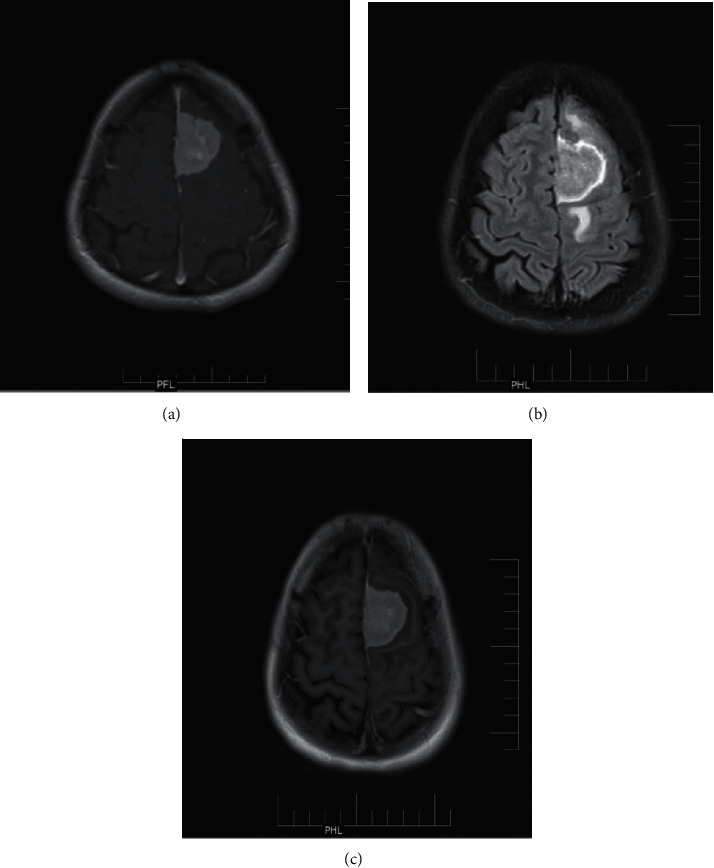
Case of meningioma: (a) postcontrast T1 MTC image; (b) delayed postcontrast T2 FLAIR image; (c) delayed postcontrast T1 FLAIR image.

Out of the 11 cases of meningioma, brighter enhancement was seen on delayed postcontrast T2 FLAIR images as compared to postcontrast T1 MTC images. Dural tail was easier to identify on delayed postcontrast T2 FLAIR images in 8 out of 11 cases due to brighter enhancement as compared to postcontrast T1 MTC images. In one case of meningioma, dural tail was less well appreciated than in postcontrast T1 MTC images. CSF cleft was better appreciated on postcontrast T2 FLAIR images than on postcontrast T1 MTC images in 8 out of 11 cases (Figure 3).

Dural tail and extraaxial nature of the lesion was better appreciated on postcontrast T2 FLAIR images than postcontrast T1 MTC images.

## 4. Discussion

**Figure 4:**
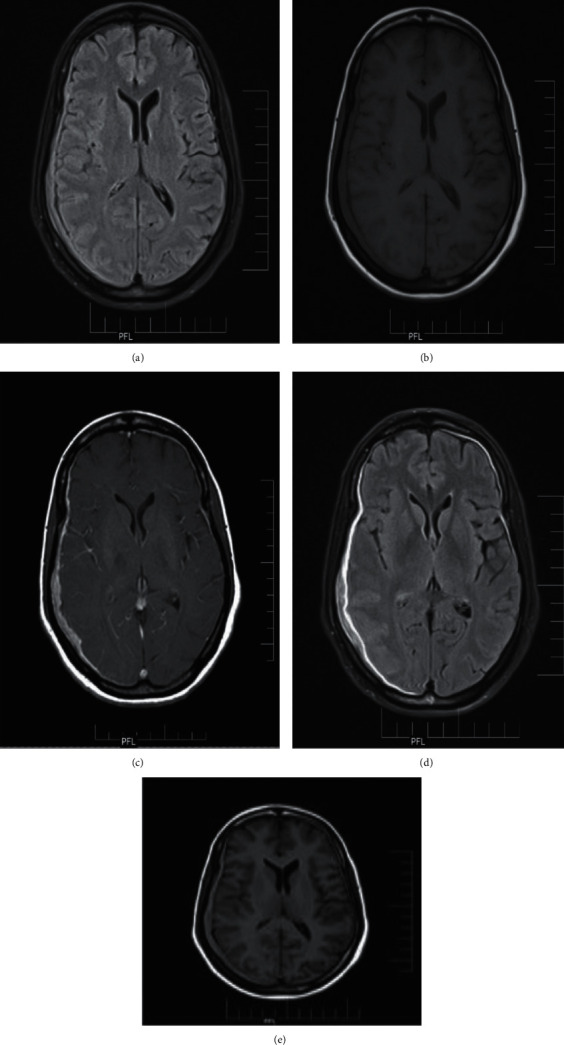
Case of meningeal metastasis: (a) unenhanced FLAIR image; (b) unenhanced T1 image; (c) postcontrast T1 MTC images; (d) delayed postcontrast T2 FLAIR image; (e) delayed postcontrast T1 FLAIR image.

Our results are similar to a report published by Kremer et al. [6] which compared unenhanced, enhanced, and delayed enhanced T2 FLAIR images with enhanced T1 MTC images in 10 patients and concluded that delayed enhanced T2 FLAIR MR sequence seems to improve the detection of lesions. The main reason for better detection of meningeal enhancement on postcontrast T2 FLAIR images is lack of vascular enhancement [7] unlike postcontrast T1 MTC images, where it becomes difficult to differentiate between the vascular enhancement of congested slow flowing vessels from meningeal enhancement (Figure 4).

Parmar et al. [8] in an in vitro phantom study showed that FLAIR was more sensitive to gadolinium effects at four times lower concentrations than T1-weighted images.

Studies done by Splendiani et al. and Artzi et al. [9] show similar results with overall sensitivity of enhanced FLAIR sequence to be 100% and 85%, respectively.

We have found that, in delayed postcontrast T1 and T2 FLAIR images, most of the intra-axial parenchyma lesions appeared more conspicuous or similar to T1 MTC images. Flair images show increased signal intensity because the T1 and T2 effects of various tissues supplement each other.

In a study done by Hakyemez et al. [10] in patients with glioblastoma multiforme, nonenhancing FLAIR hyperintense areas surrounding the enhancing lesion represented vasogenic edema, and further evaluation of this nonenhancing FLAIR hyperintense area was done with perfusion and spectroscopy to rule out tumor infiltration.

However, in our study, we found that delayed postcontrast FLAIR images can be helpful in differentiating between metastatic disease and high-grade glioma as a greater degree of perilesional signal change representing edema is more indicative of metastatic disease, as described in another study [11].

In our study, we found that delayed postcontrast T1 FLAIR images were not sensitive for detection of leptomeningeal enhancement. Any leak of gadolinium into the CSF space may shorten the TI of CSF enough so that the longitudinal magnetization recovers before the null point of CSF on T2 FLAIR images; however, this leak of gadolinium is not enough for the recovery of CSF signal intensity on T1 FLAIR images due to the shorter TI time and more T1 weightage of the images as compared to T2 FLAIR images.

Postcontrast T1 FLAIR images provided better anatomic delineation, with a better contrast between the gray and white matter and also between gray matter and the lesion, and T2 FLAIR showed delineation between lesion and CSF due to the darker CSF on T2 FLAIR images.

**Figure 5:**
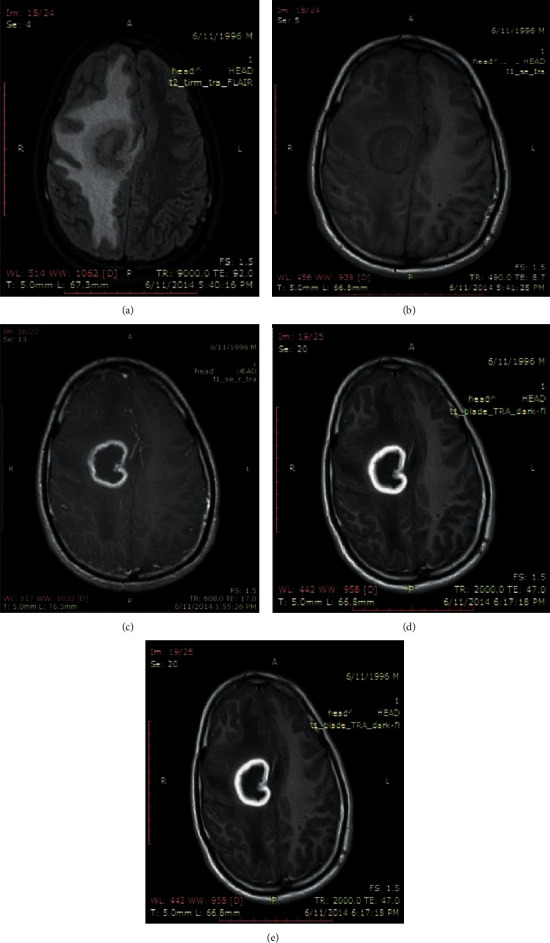
Ring enhancing lesion-tuberculoma: (a) unenhanced FLAIR image; (b) unenhanced T1 image; (c) postcontrast T1 MTC image; (d) delayed postcontrast T1 FLAIR image; (e) delayed postcontrast T1 Flair.

We found that delayed postcontrast T1 FLAIR images provided better anatomic delineation with intense enhancement of high-grade tumors, large metastatic, and infectious ring-enhancing lesions (Figure 5).

Thickened walls of chronic lesions have increased vascularity which leads to accumulation of gadolinium within the lesions, and high-grade tumors with increased perfusion show increased gadolinium retention in blood pool or within the tumor. Retention of gadolinium results in decrease of TI in CSF so that it is no longer nulled by the inversion pulse and appears bright. The cystic nonenhancing components within the lesion appear even more hypointense (than on T1 MTC) due to the inversion pulse.

Increased contrast between enhancing hyperintense and nonenhancing cystic component of the lesions along with the intrinsic high CNR of lesions on T1 FLAIR images provides excellent delineation of lesions on delayed postcontrast T1 FLAIR images. Our findings were consistent with those of Al-Saeed et al. [12].

**Figure 6:**
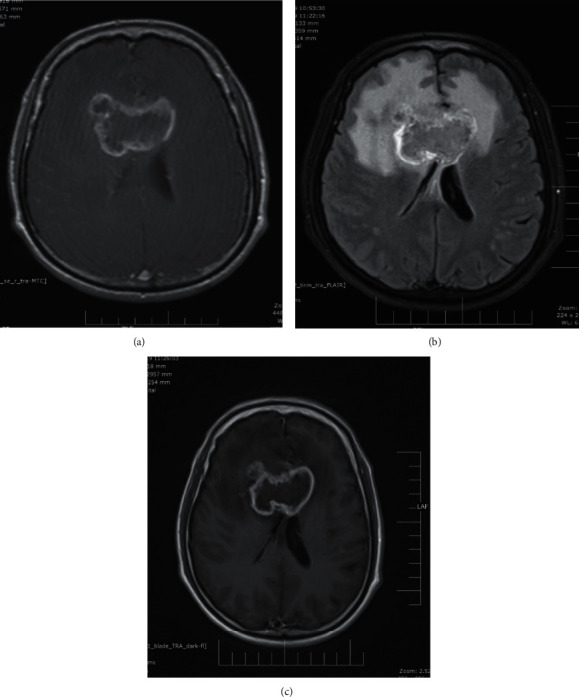
Butterfly glioma: (a) T1 MTC postcontrast; (b) T2 FLAIR postcontrast; (c) TI FLAIR postcontrast.

The overall image contrast was judged to be superior on T1-weighted FLAIR images compared with T1-weighted FSE images by all neuroradiologists [12] (Figure 6).

Delayed postcontrast T2 and T1 FLAIR images showed better enhancement of meningeal and parenchymal lesions and detected more number of lesions as compared to postcontrast T1 MTC. Delayed postcontrast T2 FLAIR images provided better delineation of extraaxial mass lesions with more conspicuous enhancement and better demarcation of dural tail.

Hence, we concluded that delayed postcost contrast T2 FLAIR images should be included in the routine protocol for contrast MRI brain study.

On the contrary, postcontrast T1 FLAIR had no added advantage over T1 MTC images in detection of meningeal lesions. Although T1 FLAIR images provided better delineation of the extraaxial mass lesions, the degree of enhancement was either inferior or similar to T1 MTC images.

Postcontrast T1 FLAIR provides better enhancement and delineation of intraparenchymal high-grade vascular tumors and large tuberculomas than postcontrast T1 MTC.

## Data Availability

The data used to support the findings of this study are included within the article.
